# Supplementary material for: Discovery of high-performance low-cost n-type Mg3Sb2-based thermoelectric materials with multi-valley conduction bands
Source: Nat Commun. 2017 Jan 6;8:13901. doi: 10.1038/ncomms13901 (PMC5227096; doi:10.1038/ncomms13901)
Supplement: Supplementary Information — Supplementary Figures, Supplementary Table, Supplementary Notes and Supplementary References. [file ncomms13901-s1.pdf]

Supplementary Figures

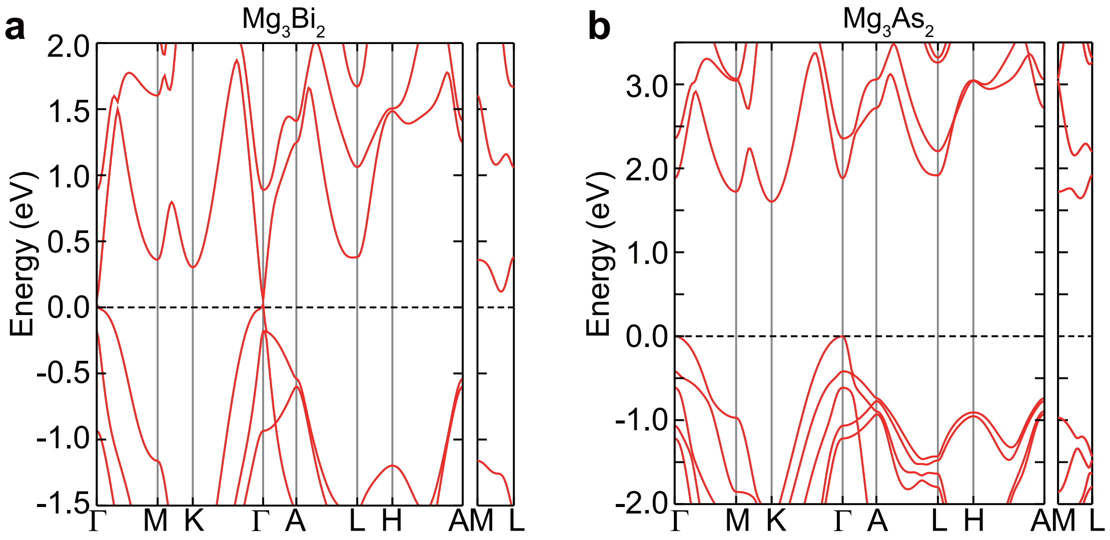

Supplementary Figure 1. Band structures of (a) Mg_3Bi_2 and (b) Mg_3As_2 including spin orbit coupling.

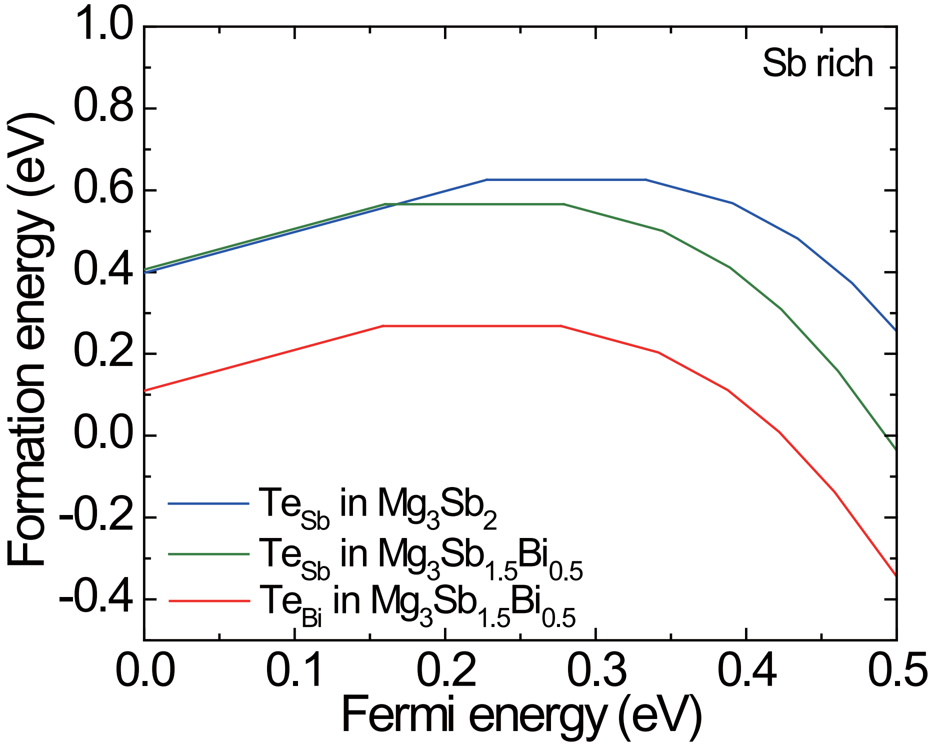

Supplementary Figure 2. Calculated formation energy as a function of Fermi energy for Te doping on the anion sites of Mg_3Sb_2 and $Mg_3Sb_{1.5}Bi_{0.5}$. The formation energies are calculated under the Sb-rich condition with $\Delta\mu_{Sb} = 0$.

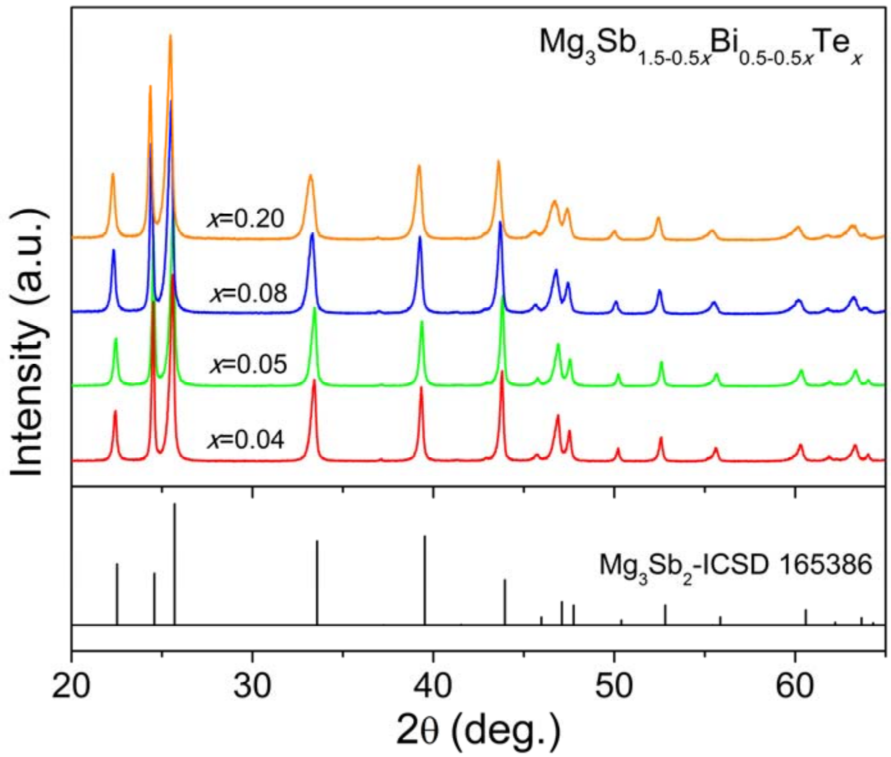

Supplementary Figure 3. X-Ray diffraction patterns of n-type Te-doped $Mg_3Sb_{1.5}Bi_{0.5}$ pellets with nominal compositions $Mg_3Sb_{1.5-0.5x}Bi_{0.5-0.5x}Te_x$ (x = 0.04, 0.05, 0.08, and 0.20).

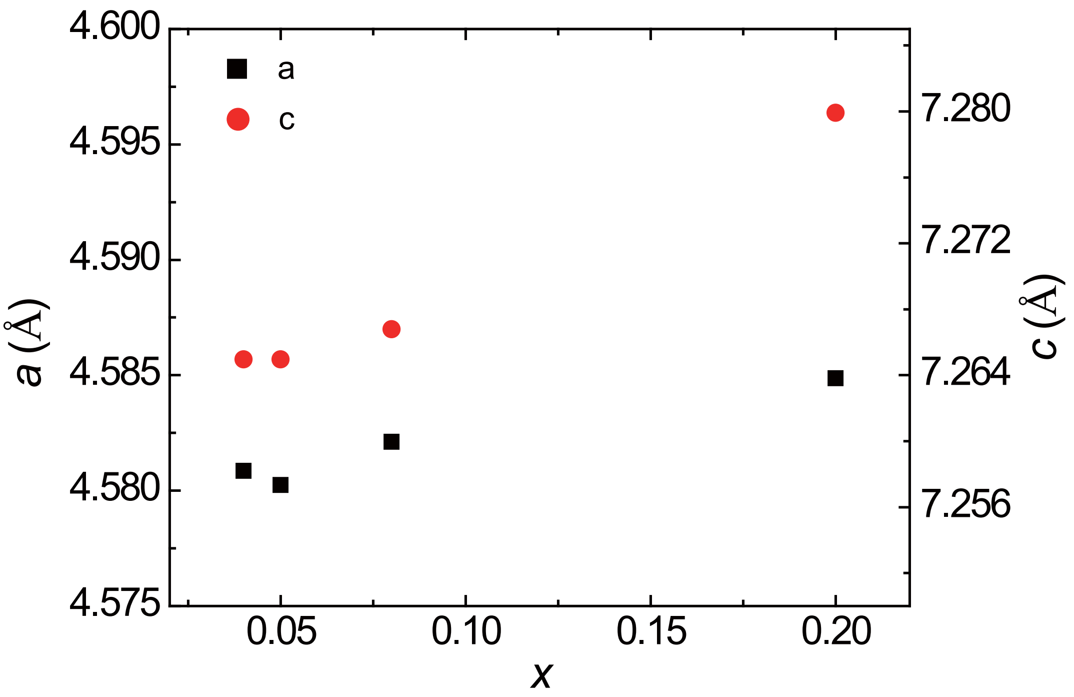

Supplementary Figure 4. Lattice constants of $Mg_3Sb_{1.5-0.5x}Bi_{0.5-0.5x}Te_x$ (x = 0.04, 0.05, 0.08, and 0.20) as a function of the fraction x.

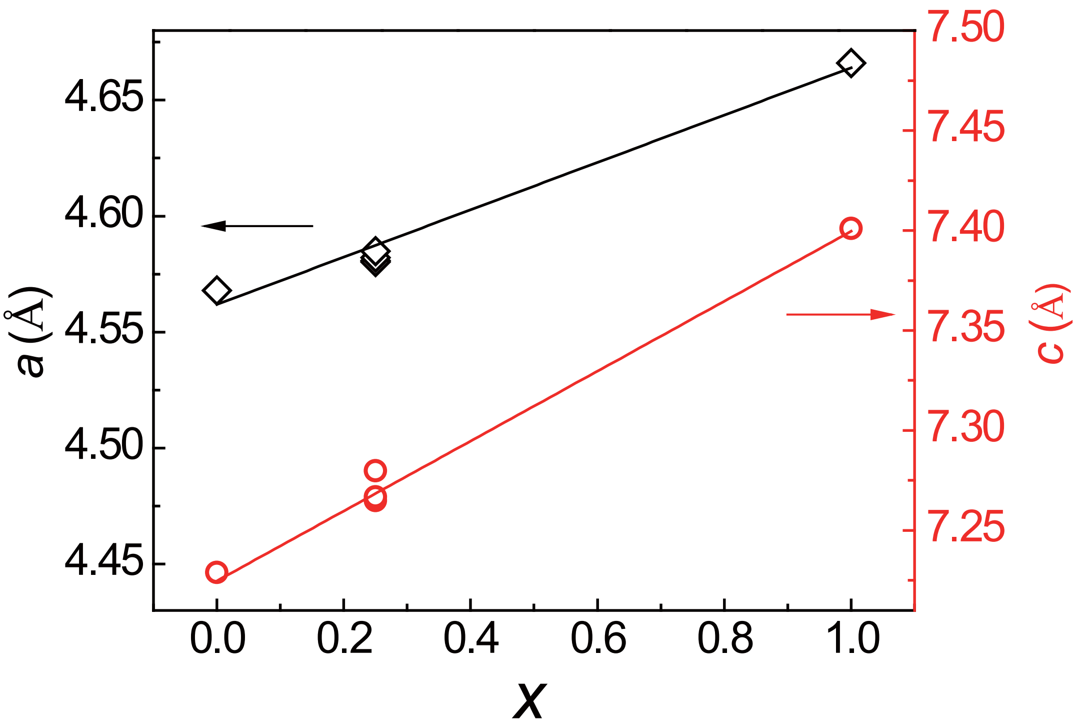

Supplementary Figure 5. Lattice constants of all Te-doped $Mg_3(Sb_{1-x}Bi_x)_2$ (x = 0.25) samples. Experimental lattice constants of Mg_3Sb_2 (x = 0) and Mg_3Bi_2 (x = 1) are taken from ICSD database.

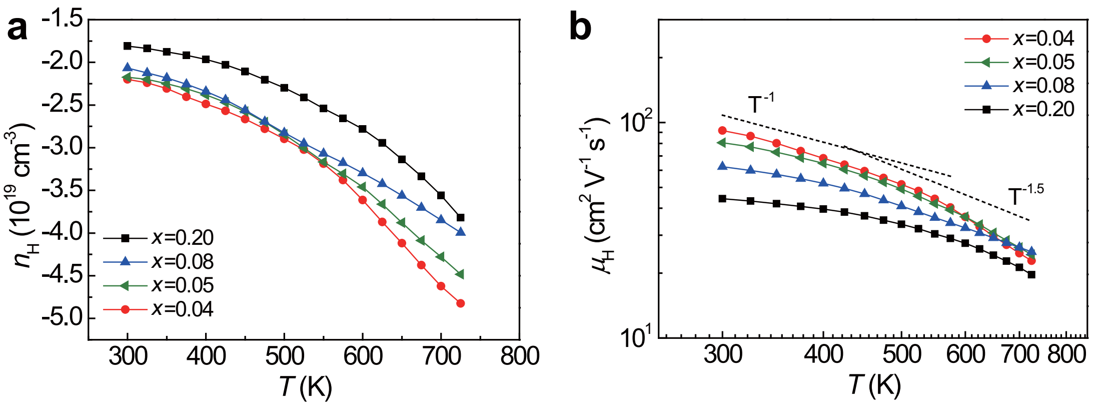

Supplementary Figure 6. Temperature dependence of (a) Hall carrier concentration n_H and (b) Hall mobility μ_H in n-type $Mg_3Sb_{1.5-0.5x}Bi_{0.5-0.5x}Te_x$. Hall mobility of $Mg_3Sb_{1.5-0.5x}Bi_{0.5-0.5x}Te_x$ (x = 0.04, 0.05, and 0.08) roughly follows $\mu \propto T^{-p}$ $(1 \leq p \leq 1.5)$ relation, indicating acoustic phonon scattering behavior.

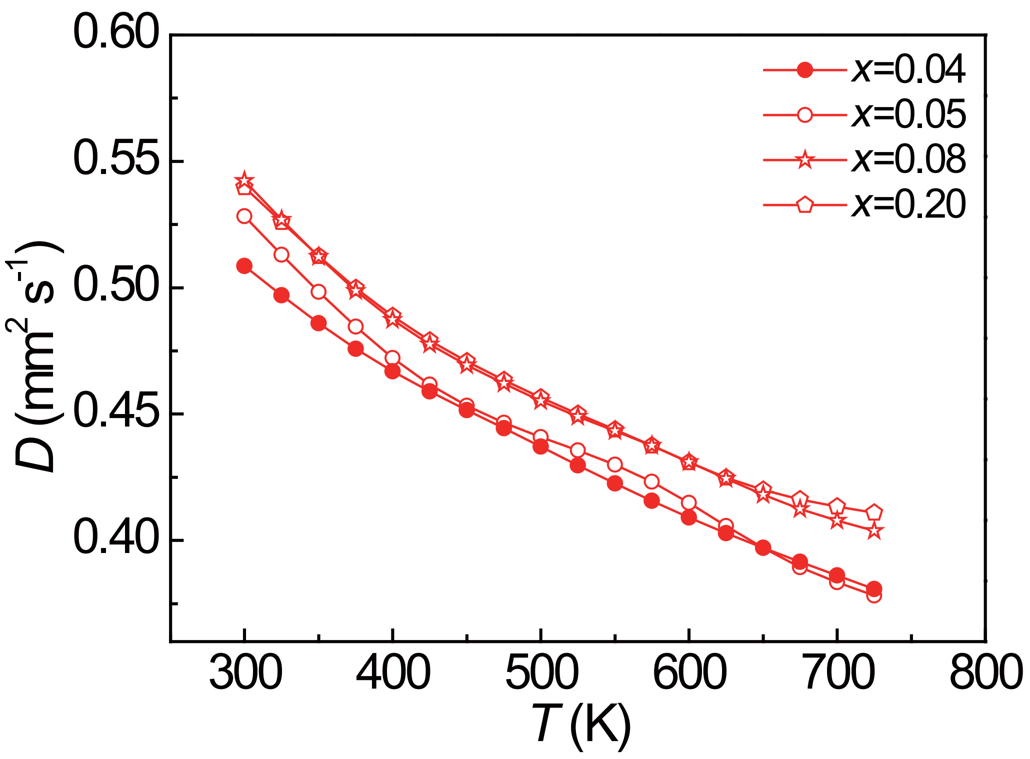

Supplementary Figure 7. Temperature dependence of thermal diffusivity in n-type $Mg_3Sb_{1.5-0.5x}Bi_{0.5-0.5x}Te_x$ (x = 0.04, 0.05, 0.08, and 0.20).

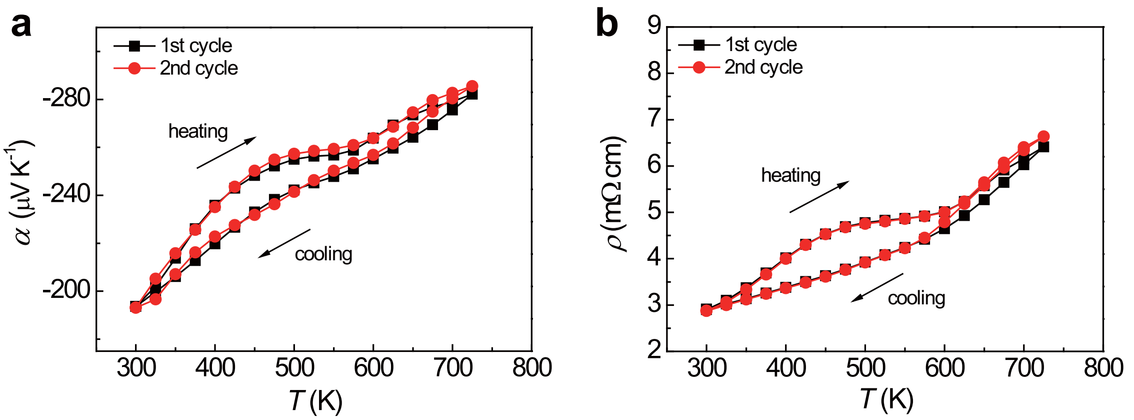

Supplementary Figure 8. Temperature dependence of (a) Seebeck coefficient and (b) the resistivity with both heating and cooling curves of the final two cycles transport measurements by ZEM-3 setup in $Mg_3Sb_{1.5-0.5x}Bi_{0.5-0.5x}Te_x$ (x = 0.05).

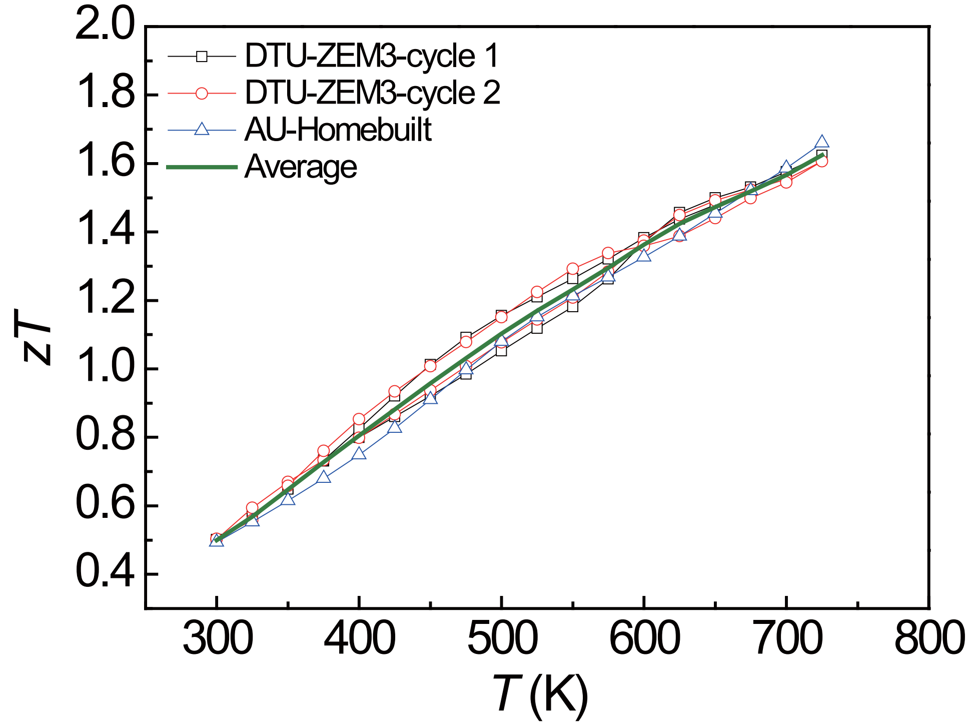

Supplementary Figure 9. Temperature-dependent zT of the high-performance sample with $x = 0.05$ obtained by the ZEM-3 setup at Technical University of Denmark (DTU) and the home-built system at Aarhus University (AU).

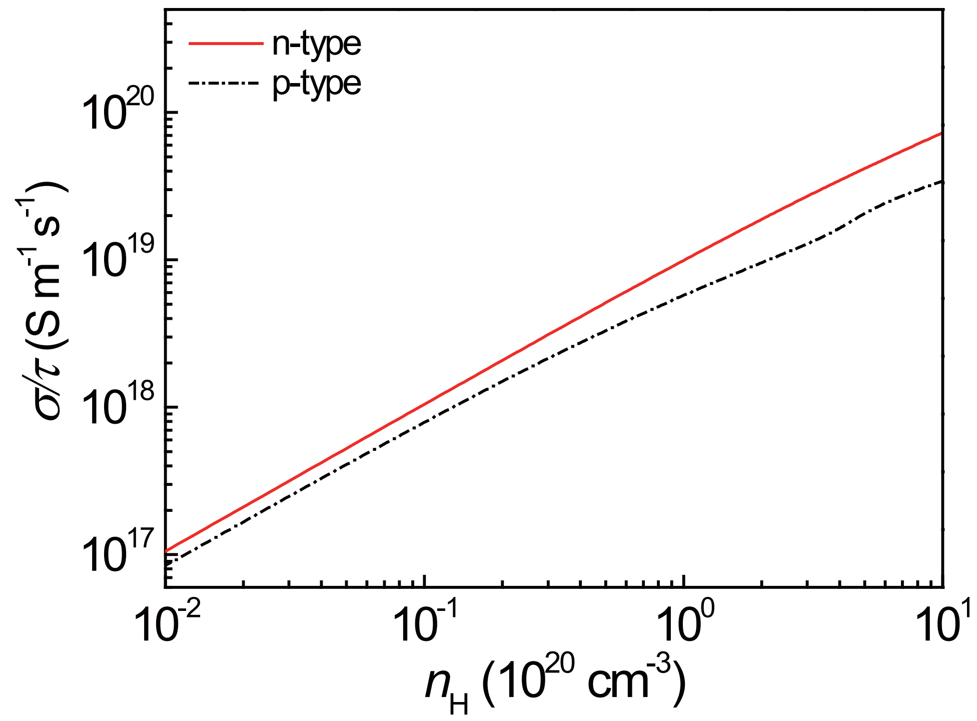

Supplementary Figure 10. Theoretical electrical conductivity σ/τ as a function of Hall carrier concentration in p-type and n-type Mg_3Sb_2. τ is the constant carrier's scattering time.

Supplementary Tables

Supplementary Table 1. The actual composition estimated by SEM-EDS analysis for the high-performance $Mg_3Sb_{1.5-0.5x}Bi_{0.5-0.5x}Te_x$ sample with x = 0.05. The actual composition by SEM-EDS is close to the nominal composition. The actual composition shows less Bi and more Sb relative to the nominal composition. This is possibly because that more Te has been doped on the Bi sites than on the Sb sites due to the lower formation energy of Te_{Bi} (see Supplementary Fig. 2).

Atom % of Nominal Composition	Atom % by SEM-EDS
$Mg_{60}Sb_{29.5}Bi_{9.5}Te_{1.0}$	$Mg_{58.9}Sb_{32.0}Bi_{7.9}Te_{1.2}$

Supplementary Notes

Supplementary Note 1. Structural Characterization

Supplementary Figure 3 shows the X-ray diffraction results of n-type Te-doped $Mg_3Sb_{1.5}Bi_{0.5}$ pellets with nominal compositions $Mg_3Sb_{1.5-0.5x}Bi_{0.5-0.5x}Te_x$ (x = 0.04, 0.05, 0.08, and 0.20). All major diffraction peaks are indexed as the Mg_3Sb_2 phase (Space Group $P\bar{3}m1$), as identified by Powder Diffraction File reference number 165386 in ICSD database. The lattice parameters of all Te-doped $Mg_3Sb_{1.5}Bi_{0.5}$ samples (Space Group $P\bar{3}m1$) shown in Supplementary Fig. 4 are determined by the Rietveld refinement method using the FullProf software[1]. As shown in Supplementary Fig. 5, the lattice parameters of Te-doped $Mg_3Sb_{1.5}Bi_{0.5}$ follow the linear curve between Mg_3Sb_2 and Mg_3Bi_2, obeying well the Vegard's law. This indicates that Te-doped $Mg_3Sb_{1.5}Bi_{0.5}$ solid solution is formed.

Supplementary Note 2. Electrical transport measurements

Electrical transport properties of Te-doped $Mg_3Sb_{1.5}Bi_{0.5}$ show small hysteresis during heating and cooling. Both heating and cooling curves of electrical transport properties of $Mg_3Sb_{1.5-0.5x}Bi_{0.5-0.5x}Te_x$ (x = 0.05) of the final two cycles measured by ZEM-3 setup are shown in Supplementary Fig. 8. The transport properties of the two cycles are consistent upon the repeated heating and cooling measurements. In addition, the thermoelectric zT, obtained on the high-performance sample with x = 0.05 by the home-built system and ZEM-3 setup, shows excellent consistency between each other (Supplementary Fig. 9). The good repeatability and reproducibility observed in different setups indicate that the hysteresis in the sample is more likely caused by reversible processes. The obtained zT values by the heating and cooling curve are comparable (Supplementary Fig. 9), thus the experimental data measured during cooling are used in the main text.

Supplementary Note 3. Electrical transport calculations

Electrical transport properties in the main text are simulated using combined full band structure calculation and semi-classical Boltzmann transport theory under the constant

scattering time approximation (CSTA) as implemented in the BoltzTraP code[2]. Under the CSTA, it is assumed that the carrier scattering time τ determining the electrical conductivity will not vary strongly with temperature and doping level. From *ab initio* full band structures, the group velocity of an electron in a specific band can be calculated by:

$$\mathbf{v}_{\alpha}(i,\mathbf{k}) = \frac{1}{\hbar}\frac{\partial \varepsilon_{i,\mathbf{k}}}{\partial \mathbf{k}_{\alpha}} \quad (1)$$

where $\varepsilon_{i,\mathbf{k}}$ is the ith energy band at point $\mathbf{k}$ and $\mathbf{k}_{\alpha}$ is αth component of wave vector $\mathbf{k}$.

Using the group velocity $\mathbf{v}_{\alpha}(i, \mathbf{k})$, the electrical conductivity and Seebeck coefficient tensors can then be calculated by the following equations:

$$\sigma_{\alpha\beta}(T,\mu) = \frac{1}{\Omega}\int \bar{\sigma}_{\alpha\beta}(\varepsilon)\left[-\frac{\partial f_0(T,\varepsilon,\mu)}{\partial \varepsilon}\right]d\varepsilon \quad (2)$$

$$S_{\alpha\beta}(T,\mu) = \frac{1}{eT\Omega\sigma_{\alpha\beta}(T,\mu)}\int \bar{\sigma}_{\alpha\beta}(\varepsilon)(\varepsilon-\mu)\left[-\frac{\partial f_0(T,\varepsilon,\mu)}{\partial \varepsilon}\right]d\varepsilon \quad (3)$$

where $\bar{\sigma}_{\alpha\beta}(\varepsilon)$ can be expressed as:

$$\bar{\sigma}_{\alpha\beta}(\varepsilon) = \frac{e^2}{N}\sum_{i,\mathbf{k}} \tau_{i,\mathbf{k}} \cdot v_{\alpha}(i,\mathbf{k}) \cdot v_{\beta}(i,\mathbf{k}) \cdot \frac{\delta(\varepsilon-\varepsilon_{i,\mathbf{k}})}{d\varepsilon}. \quad (4)$$

$\tau_{i,\mathbf{k}}$ is the relaxation time, α and β are the Cartesian indices, N is the total number of $\mathbf{k}$-points sampled, Ω is the volume of the unit cell, $f_0(T,\varepsilon,\mu)$ is the Fermi Dirac distribution function, μ is the chemical potential, e is the elementary charge and T is temperature.

Although the electrical conductivity and power factor are calculated with respect to the constant scattering time τ, the calculated Seebeck coefficient is independent of τ. This approach has been successfully applied to predict the Seebeck coefficient and the trend of the electrical conductivity or power factor for a variety of thermoelectric materials[3-6]. The experimental data of p-type doped Mg_3Sb_2 agrees very well with the curve simulated by semi-classical Boltzmann transport theory under CSTA shown in Fig. 2a in the main text, proving the validity of this approach. The calculated electrical conductivity of n-type doping is higher than that of p-type doping in Mg_3Sb_2 (Supplementary Fig. 10), agrees very well with the experimental result.

Ab initio band structure of Mg_3Sb_2 shows parabolic-like band dispersions at the band

edges along different high symmetry lines, making it possible to analyze the contributions from the different bands by a single parabolic band model. By fitting the *ab initio* band structure, the effective mass tensor m_{ij}^* of a single valley can be calculated by

$$m_{ij}^* = \hbar^2[\partial^2\varepsilon(k)/\partial k_i\partial k_j]^{-1}. \quad (5)$$

The single valley effective mass then can be obtained by

$$m_{\mathrm{s}}^* = (m_{\mathrm{xx}}^* m_{\mathrm{yy}}^* m_{\mathrm{zz}}^*)^{1/3}. \quad (6)$$

The density of states (DOS) effective mass can be calculated by

$$m_{\mathrm{d}}^* = N_{\mathrm{v}}^{2/3} m_{\mathrm{s}}^*. \quad (7)$$

where N_{v} is band valley degeneracy.

Under a single parabolic band approximation, the Seebeck coefficient, Hall carrier concentration and Hall factor can be written as[7]

$$S = \mp\frac{k_{\mathrm{B}}}{e}\left(\eta - \frac{\left(r+\frac{5}{2}\right)F_{r+3/2}(\eta)}{\left(r+\frac{3}{2}\right)F_{r+1/2}(\eta)}\right) \quad (8)$$

$$n_{\mathrm{H}} = 4\pi\left(\frac{2m^* k_{\mathrm{B}}T}{h^2}\right)^{3/2}\frac{F_{1/2}(\eta)}{r_{\mathrm{H}}} \quad (9)$$

$$r_{\mathrm{H}} = \frac{3}{2}F_{1/2}(\eta)\frac{(3/2+2r)F_{2r+1/2}(\eta)}{(3/2+r)^2 F_{r+1/2}^2(\eta)}, \quad (10)$$

where the Fermi integral is

$$F_n(\eta) = \int_0^\infty \frac{\varepsilon^n d\varepsilon}{1+\exp(\varepsilon-\eta)}, \quad (11)$$

k_{B} is the Boltzmann constant, h is the Planck's constant, η is the reduced Fermi energy. r=-0.5 for acoustic phonon scattering is assumed.

The effective masses of the band edges are anisotropic in Mg_3Sb_2, in agreement with the 2D layered structure. The effective masses of the K conduction band ($N_{\mathrm{v}} = 2$) are calculated as m_{xx}*=m_{yy}*=0.32m_{e} and m_{zz}*=0.21m_{e}, which yield m_{s}*=0.28m_{e} and m_{d}*=0.45m_{e}. For the ML conduction band ($N_{\mathrm{v}} = 6$) along M-L line, m_{xx}*=0.55m_{e}, m_{yy}*=0.21m_{e}, and m_{zz}*=0.28m_{e}, resulting in m_{s}*=0.32m_{e} and m_{d}*=1.05m_{e}. For the Γ valence band ($N_{\mathrm{v}} = 1$), m_{xx}*=m_{yy}*=1.15m_{e}

and m_{zz}*=0.15m_e, leading to m_s*=0.58m_e and m_d*=0.58m_e. From the above results, it is clear that the ML band and the Γ band are anisotropic, which is confirmed by the shapes of Fermi surfaces shown in Fig. 2d,e. It should be noted that the single parabolic band model used here will cause some uncertainties due to the anisotropic band features. In this work, the single parabolic band model is only used to compare with the accurate semi-classical Boltzman transport theory to understand the multiple band behavior.

The experimental DOS effective mass can be obtained by combining the above single band model formulas and experimental data including Seebeck coefficient, Hall carrier concentration, and temperature under the acoustic phonon scattering mechanism. The discussion in Fig. 3d in the main text is based on the assumption that the band structure will not vary strongly with increasing temperature.

Supplementary Note 4. Estimation of the carrier's scattering time

The carrier's scattering time τ is estimated from the relation $\mu = e\tau / m_{\mathrm{I}}^{*}$, where μ is the carrier mobility, m_{I}^{*} is inertial or conductivity effective mass, and e is the elementary charge. m_{I}^{*} can be expressed as

$$m_{\mathrm{I}}^{*} = 3 / (2 / m_{\mathrm{xx}}^{*} + 1 / m_{\mathrm{zz}}^{*}) \qquad (12)$$

m_{I}^{*} of the near-edge Γ valence band is calculated as 0.36m$_e$. Combining experimentally reported mobility[8] μ = 16 cm^2 V^{-1} s^{-1} at room temperature, we can obtain the constant carrier scattering time τ = 3.25×10^{-15} s for p-type Mg_3Sb_2. For n-type transport in Mg_3Sb_2, since the ML band and the K band are nearly converged, we use the average of conductivity mass of two bands as

$$m_{\mathrm{I}}^{*} = 2 / (1 / m_{\mathrm{I,K}}^{*} + 1 / m_{\mathrm{I,ML}}^{*}) \, . \qquad (13)$$

m_{I}^{*} of n-type Mg_3Sb_2 thus is estimated as 0.28m_e. Under acoustic phonon scattering, according to the relation $\mu \propto 1/m_s^{*5/2}$ (ref. 7) and p-type mobility, the mobility of n-type Mg_3Sb_2 can be estimated to be 84 cm^2 V^{-1} s^{-1} using the average m_s*=0.30m_e of the K band and the ML band. Then, we can estimate the constant carrier scattering time τ = 1.34×10^{-14} s for

n-type Mg_3Sb_2.

Supplementary Note 5. Effective band structures of solid solutions by a band unfolding technique

To discuss the orbital engineering[5,6] at the Γ point in solid solutions it is usually correct to directly use band structures from a supercell calculations since normally at the center of the Brillouin zone Γ point the band structure will not get folded at the valence band maximum. However, studying the multiple conduction bands of solid solutions by supercell calculation will always have zone folding problems, since multiple conduction bands normally locate at relatively low symmetry k point. Thus, unfolding the band structure of a supercell to recover a primitive cell picture is desired. Here we carry out the band unfolding using the spectral weight approach. In this approach, we need to construct a spectral function $A(\mathbf{k}, E)$, given by[9-11]

$$A(\mathbf{k},E)=\sum_m P_{\mathbf{K}m}(\mathbf{k})\delta(E_m-E)\,, \tag{14}$$

where E is the continuously variable energy and $P_{\mathbf{K}\mathrm{m}}(\mathbf{k})$ is the spectral weight, expressed as

$$P_{\mathbf{K}m}=\sum_n \left|\langle \mathbf{K}m|\mathbf{k}n\rangle\right|^2\,, \tag{15}$$

where the $|\mathbf{k}n\rangle$ and $|\mathbf{K}m\rangle$ (n and m are band indices) are respectively the eigenvectors in the primitive cell and the supercell. The spectral weight can be obtained by projecting $|\mathbf{K}m\rangle$ on all primitive cell eigenstates $|\mathbf{k_i}n\rangle$ of a fixed $\mathbf{k_i}$. Thus, $A(\mathbf{k}, E)$ can be obtained, which is regarded as an effective primitive cell projection of the supercell band structure. The detailed description of the effective band unfolding method is shown in refs 9-11.

Supplementary Note 6. The calculation of defect formation energy

The defect calculations were conducted in a 3×3×2 supercell with 90 atoms using PBE functional[12] as implemented in VASP code[13]. The energy and Hellmann-Feynman force convergence criteria were 10^{-4} eV and 0.008 eV Å^{-1}, respectively. The plane-wave energy cutoff was set at 400 eV. A 3×3×3 k mesh and a 6×6×6 k mesh were applied for the crystal structure optimization and the total energy calculation, respectively. For the optimization of defect structures, the lattice parameters were fixed at the optimized values of the perfect

supercell and all the ions in the defect supercell were fully relaxed into their equilibrium positions. For simplicity, no energy corrections have been used in the defect formation energy.

The formation energy of a defect or impurity D with a charge state q is defined as:[14,15]

$$E_{\mathrm{f}}(D^q) = E_{\mathrm{tot}}(D^q) - E_{\mathrm{tot}}(bulk) - \sum_i n_i \mu_i + q(\varepsilon_{\mathrm{F}} + E_{\mathrm{V}} + \Delta V)\,. \qquad (16)$$

$E_{\mathrm{tot}}(D^q)$ is the total energy obtained from a supercell calculation with a defect D in a charge state q, and $E_{\mathrm{tot}}(bulk)$ is the total energy of the perfect supercell. n_i represents the number of atoms of type i that is added to ($n_i > 0$) or removed from ($n_i < 0$) the supercell when the defect or impurity is formed, and the μ_i indicates the atomic chemical potentials of these species. ε_{F} is the Fermi level referenced to the energy of the valence band maximum E_{V} of the perfect bulk supercell. ΔV is a correction term to align the reference potential of the defect supercell with that of the bulk supercell.

For Mg_3Sb_2, the chemical potentials must satisfy the stability condition:

$$3\Delta\mu_{\mathrm{Mg}} + 2\Delta\mu_{\mathrm{Sb}} = 5\Delta H_{\mathrm{f}}(\mathrm{Mg_3Sb_2}) \qquad (17)$$

with $\Delta\mu_{\mathrm{Mg}} \le 0$ and $\Delta\mu_{\mathrm{Sb}} \le 0$, and $\Delta H_{\mathrm{f}}(\mathrm{Mg_3Sb_2})$ is the formation enthalpy of Mg_3Sb_2. For the Te_{Sb} defect, the chemical potentials are further limited the following conditions to avoid the precipitations of MgTe, $MgTe_2$, Sb_2Te_3:

$$\Delta\mu_{\mathrm{Mg}} + \Delta\mu_{\mathrm{Te}} \le 2\Delta H_{\mathrm{f}}(\mathrm{MgTe})\,, \qquad (18)$$

$$\Delta\mu_{\mathrm{Mg}} + 2\Delta\mu_{\mathrm{Te}} \le 3\Delta H_{\mathrm{f}}(\mathrm{MgTe_2})\,, \qquad (19)$$

$$2\Delta\mu_{\mathrm{Sb}} + 3\Delta\mu_{\mathrm{Te}} \le 5\Delta H_{\mathrm{f}}(\mathrm{Sb_2Te_3})\,, \qquad (20)$$

with $\Delta\mu_{\mathrm{Te}} \le 0$. The formation enthalpies of Mg_3Sb_2, MgTe, $MgTe_2$, and Sb_2Te_3 are calculated to be -0.37, -0.88, -0.58, and -0.13 eV per atom.

Similarly, for $Mg_3Sb_{1.5}Bi_{0.5}$, the chemical potentials must satisfy the stability condition:

$$3\Delta\mu_{\mathrm{Mg}} + 1.5\Delta\mu_{\mathrm{Sb}} + 0.5\Delta\mu_{\mathrm{Bi}} = 5\Delta H_{\mathrm{f}}(\mathrm{Mg_3Sb_{1.5}Bi_{0.5}}) \qquad (21)$$

with $\Delta\mu_{\mathrm{Mg}} \le 0$, $\Delta\mu_{\mathrm{Sb}} \le 0$, and $\Delta\mu_{\mathrm{Bi}} \le 0$. For Te_{Sb} and Te_{Bi} defects, the chemical potentials are further constrained by the formation of MgTe, $MgTe_2$, Sb_2Te_3, Bi_2Te_3, Bi_4Te_3, and BiTe.

$\Delta\mu_\alpha = \mu_\alpha - \mu_\alpha^0$ (α = Mg, Sb, Bi, and Te), and the μ_α^0 indicates the total energy per atom in the pure bulk crystal of type α.

The defect formation energy data of tellurium doping on the anion sites in Mg_3Sb_2 and $Mg_3Sb_{1.5}Bi_{0.5}$ is shown in Supplementary Fig. 2. The defect formation energy is calculated under the Sb-rich condition with $\Delta\mu_{Sb} = 0$, which is based on the fact that Sb secondary phase can be easily found in the traditional synthesis[16]. As shown in Supplementary Fig. 2, the formation energy of Te_{Sb} in $Mg_3Sb_{1.5}Bi_{0.5}$ is slightly lower than that of Te_{Sb} in Mg_3Sb_2, while doping tellurium on the Bi sites in $Mg_3Sb_{1.5}Bi_{0.5}$ shows the formation energy much lower than that of Te_{Sb} defect in either $Mg_3Sb_{1.5}Bi_{0.5}$ or Mg_3Sb_2. This result indicates that Te doping on the Bi sites probably will be easier than Te doping on the Sb sites in $Mg_3Sb_{1.5}Bi_{0.5}$.

Supplementary References

1. Rodríguez-Carvajal, J. Recent advances in magnetic structure determination by neutron powder diffraction. *Physica B* **192,** 55-69 (1993).
2. Madsen, G. K. H. & Singh, D. J. BoltzTraP. A code for calculating band-structure dependent quantities. *Comput. Phys. Commun.* **175,** 67-71 (2006).
3. Madsen, G. K. H. Automated search for new thermoelectric materials: The case of LiZnSb. *J. Am. Chem. Soc.* **128,** 12140-12146 (2006).
4. Yang, J. *et al.* Evaluation of half-Heusler compounds as thermoelectric materials based on the calculated electrical transport properties. *Adv. Funct. Mater.* **18,** 2880-2888 (2008).
5. Zhang, J. *et al.* Designing high-performance layered thermoelectric materials through orbital engineering. *Nature Commun.* **7,** 10892 (2016).
6. Zhang, J. *et al.* High-performance pseudocubic thermoelectric materials from non-cubic chalcopyrite compounds. *Adv. Mater.* **26,** 3848-3853 (2014).
7. Goldsmid, H. J. *Thermoelectric Refrigeration* (Plenum, 1964).
8. Ponnambalam, V. & Morelli, D. On the thermoelectric properties of Zintl compounds $Mg_3Bi_{2-x}Pn_x$ (Pn = P and Sb). *J. Electron. Mater.* **42,** 1307-1312 (2013).
9. Medeiros, P. V. C., Stafström, S. & Björk, J. Effects of extrinsic and intrinsic perturbations on the electronic structure of graphene: Retaining an effective primitive cell band structure by band unfolding. *Phys. Rev. B* **89,** 041407 (2014).
10. Popescu, V. & Zunger, A. Extracting E versus **k** effective band structure from supercell calculations on alloys and impurities. *Phys. Rev. B* **85,** 085201 (2012).
11. Rubel, O., Bokhanchuk, A., Ahmed, S. J. & Assmann, E. Unfolding the band structure of disordered solids: From bound states to high-mobility Kane fermions. *Phys. Rev. B* **90,** 115202 (2014).
12. Perdew, J. P., Burke, K. & Ernzerhof, M. Generalized gradient approximation made simple. *Phys. Rev. Lett.* **77,** 3865-3868 (1996).
13. Kresse, G. & Furthmüller, J. Efficient iterative schemes for ab initio total-energy calculations using a plane-wave basis set. *Phys. Rev. B* **54,** 11169-11186 (1996).

14. Van de Walle, C. G. & Neugebauer, J. First-principles calculations for defects and impurities: Applications to III-nitrides. *J. Appl. Phys.* **95,** 3851-3879 (2004).

15. Zhang, S. B. & Northrup, J. E. Chemical potential dependence of defect formation energies in GaAs: Application to Ga self-diffusion. *Phys. Rev. Lett.* **67,** 2339-2342 (1991).

16. Condron, C. L., Kauzlarich, S. M., Gascoin, F. & Snyder, G. J. Thermoelectric properties and microstructure of Mg_3Sb_2. *J. Solid State Chem.* **179,** 2252-2257 (2006).
